# Cucurbit[*n*]uril-based fluorescent indicator-displacement assays for sensing organic compounds

**DOI:** 10.3389/fchem.2023.1124705

**Published:** 2023-01-13

**Authors:** Qunpeng Duan, Ran Chen, Su Deng, Cheng Yang, Xinxin Ji, Gege Qi, Hui Li, Xiaohan Li, Shihao Chen, Mengen Lou, Kui Lu

**Affiliations:** ^1^ School of Chemical and Printing-Dyeing Engineering, Henan University of Engineering, Zhengzhou, China; ^2^ School of Chemical Engineering and Food Science, Zhengzhou Institute of Technology, Zhengzhou, China

**Keywords:** cucurbit[n]urils, host-guest complex, organic compounds, fluorescence, indicator displacement assay

## Abstract

The widespread conversion of synthetic receptors into luminescent sensors has been achieved *via* the use of fluorescent-indicator displacement assays (F-IDAs). Due to their rigid structures and efficient binding affinities, cucurbit[*n*]urils, combined with a variety of fluorescent guests, have gained extensive utilization in fluorescent-indicator displacement assays for sensing non-fluorescent or weakly fluorescent organic compounds (analytes) in a selective and specific manner. This mini-review summarizes recent advances in the design of cucurbit[*n*]uril-based fluorescent-indicator displacement assays and discusses the current challenges and future prospects in this area.

## Introduction

**FIGURE 1:**
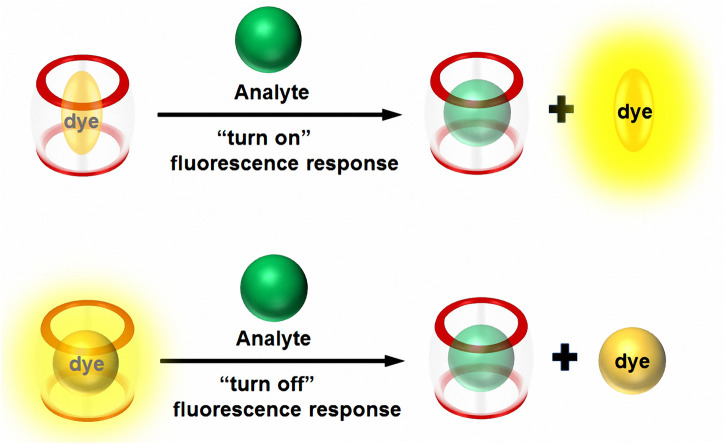
Schematic illustration of an F-IDA, where the disassembly of an integrated CB*n*-fluorophore sensor happens when an analyte exists, triggering either a “turn-on” or “turn-off” fluorescence response.

Indicator displacement assays (IDAs) (Metzger and Anslyn, 1998; Wiskur et al., 2001; Nguyen and Anslyn, 2006) have been a favored approach for achieving synthetic-receptor conversion into optical sensors in the supramolecular chemistry realm. IDAs, which exploit the non-covalent interplays of the host with analyte/indicator, have been applied to the sensing of diverse analytes (e.g., metal anions, cations, pharmaceuticals, and other biological molecules) (Rather and Ali, 2021; Sedgwick et al., 2021). IDAs are recognized as a crucial and practical strategy, especially in fluorescence-sensing applications. As the initial step of this approach, an inclusion complex is created between an appropriate indicator dye and an adequate macrocyclic host. In this way, arbitrary (mostly photophysical) traits of the dye molecules are either attenuated or enhanced. Upon application of this inclusion complex for the analyte detection in the solution, the dye’s modulated traits are reversed since the dye is displaced from the host cavity through competitive analyte–host binding (Bakirci and Nau, 2006; Nguyen and Anslyn, 2006; Praetorius et al., 2008; Dsouza et al., 2011; Jadhav et al., 2015; Norouzy et al., 2015; Sayed et al., 2016; Sayed and Pal, 2016). The utilization of fluorescent dyes in such IDAs is often regarded as profoundly useful in view of the appreciable system response to the analytes under these scenarios *via* either fluorescence “turn-off” or “turn-on” sequences (see Figure 1). This approach is defined uniquely as Fluorescent-IDAs (F-IDAs).

Behrend was the original discoverer of cucurbit[*n*]urils (CB*n*, *n* = 6–8), the third class of macrocycles, in 1905 (Behrend et al., 1905). The CB*n*, consisting of *n*-glycoluril units that are bridged by 2*n*-methylene groups, in particular, hold immense negative charge density in the carbonyl rim portals, allowing neutral and cationic organic guests to be bound. At the same time, the interior cavities of CB*n* are hydrophobic and preferably accommodate hydrophobic moieties (Isaacs, 2014). As the fluorescent guests’ photophysical traits are alterable by the CB*n* hosts (Dsouza et al., 2011), the application of CB*n* for host–guest complex creation has been reported, and these complexes have gained further utilization for sensing or determining non-fluorescent or weakly fluorescent organic compounds (analytes) *via* F-IDAs (Ghale and Nau, 2014).

**FIGURE 2:**
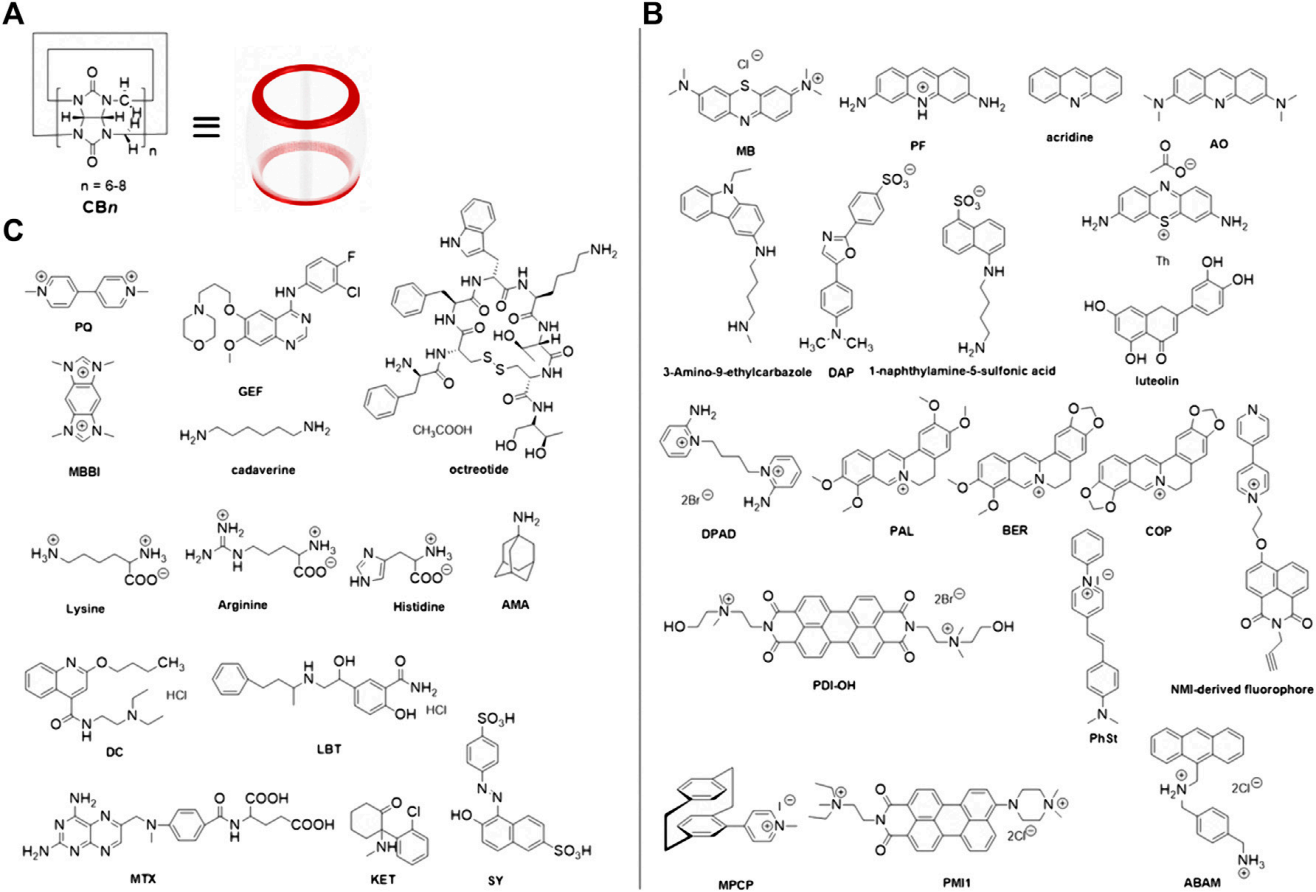
Chemical architectures for **(A)** CB*n* synthetic receptors, **(B)** fluorophore indicators, and **(C)** selected analyte competitors.

In our current mini-review, we detail recent advances in the application of a variety of CB*n* analytically applied to achieve the fluorescence intensity quenching or enhancement for various guest molecules and the design of F-IDAs induced by CB*n* based on the quenching or enhancement of fluorescence signals for the analyte assessment purpose. Figure 2 illustrates the structures of CB*n,* analytes, and fluorophores adopted in F-IDAs as synthetic receptors, competitors, or indicators.

## IDAs for analyte sensing using a “turn-on” fluorescence response

Fluorescence “turn-on” displacement assays exploit the guest molecule-induced competitive displacement of a fluorescent dye [e.g., acridine (Ac), methylene blue (MB), or proflavine (PF)] from the hydrophobic cavity of the CB*n*.

In 2008, Praetorius et al. (2008) proposed a concept of product-selective supramolecular tandem assay through F-IDA based on CB6 and diamino-alkyl-anchored 3-amino-9-ethylcarbazole as a fluorescent guest, with which amino acids and their decarboxylases were detected. Later, in 2014, Sun and co-workers employed the F-IDA strategy to detect paraquat (PQ), an environmental pollutant, where the host–guest complexes of MB and CB8 were utilized (Sun et al., 2014). MB forms a powerful inclusion complex (2:1) with CB8 in an aqueous solution, MB_2_⊂CB8. Despite the potent fluorescent trait of monomeric MB, the dimeric MB in MB_2_⊂CB8 is relatively non-fluorescent. When PQ is resent, there is a displacement of probe MB from the MB_2_⊂CB8 complex, as the competitive binding interaction of PQ with the CB8 host is more powerful. In response to the existence of PQ, MB is discharged, resulting in an immensely-enhanced fluorescence.

Xi et al. (2017) reported an innovative F-IDA for sensing an anticarcinogen known as gefitinib (GEF), where the macrocyclic host was CB8 and the fluorescent guest was proflavine (PF). They observed that a 1:2 host–guest complex was created between CB8 and PF, with the latter showing quenched fluorescence intensity. Upon incorporation of GEF into the CB8–PF mixture, the fluorescence intensity recovered as the PF was displaced by the GEF. The proposed F-IDA has demonstrated encouraging results in actual applications in determining GEF in living cells using an appropriate imaging technique. In 2018, Hirani et al. (2018) reported F-IDA based on CB8 and tetramethylbenzobis(imidazolium) (MBBI) as the fluorescent indicator for identifying non-aromatic and methionine-terminated aromatic peptides in aqueous solutions in a selective manner. It was revealed that no aromatic residue is necessary with the CB*n*-mediated identification of peptides to attain high affinity. Capable of targeting disorderly folded protein loops and peptide *N*-termini, the CB*n* are encouraging receptors for the newly translated and unmodified proteins. Similarly, F-IDA was also used in the identification and testing of octreotide based on CB8 and acridine as an F-IDA system (Yin et al., 2019).

## IDAs for analyte sensing using a “turn-off” fluorescence response

Fluorescence “Turn-off” displacement assays mainly exploit the guest molecule-induced competitive displacement of a fluorescent dye [e.g., dapoxyl (DAP), acridine orange (AO), palmatine (PAL), berberine (BER), or coptisine (COP)] from the CB*n* hydrophobic cavity.

In 2007, Hennig et al. (2007) reported an F-IDA for the surveillance of cationic products from reactions catalyzed by the amino acid decarboxylases. CB7 was used to interact with DAP as a fluorescent guest. DAP exhibited a 200-fold enhancement in its fluorescence intensity after binding to CB7, as well as a turn-off of signal following ammonium product-induced displacement. Given the stronger affinity of CB7 for cationic alkylammonium products than amino acids, the amino acid incorporation failed to interfere with the F-IDA. In another study, a label-free F-IDA strategy was designed by Nau and co-workers to achieve real-time continuous protease activity surveillance on unlabeled peptides, where CB7 and AO were utilized as the macrocyclic host and fluorescent guest, respectively (Ghale et al., 2011). CB7 was responsible for selectively identifying the cleavage products, which bear an N-terminal phenylalanine residue. AO was responsible for the reaction signaling after being selectively displaced from CB7 by the high-affinity proteolysis product. An established inhibitor phosphoramidon was adopted to verify the protease inhibition quantifying the capacity of the proposed F-IDA strategy. Wang et al. (2014) employed another label-free competitive fluorescence displacement system comprising the macrocyclic host CB7, and AO was employed for the detection of cadaverine. Following the incorporation of the cadaverine into a strongly fluorescent supramolecular CB7–AO complex, the dye was displaced, and the fluorescence was attenuated. For the activity surveillance of lysine decarboxylase (an enzyme enabling lysine conversion into cadaverine), differential fluorescence responses were observed for the enzymatic reactant, as well as its product. Enzyme suppression by different organophosphate esters was assessed using the proposed system. In 2011, an extraordinary F-IDA was put forward by Florea and Nau (2011), which enabled volatile hydrocarbon surveillance in aqueous solutions in real time. CB6 was employed as the host, and putrescine-anchored 1-naphthylamine-5-sulfonic acid was adopted as the fluorescent guest. After an aqueous solution of the F-IDA was exposed to diverse hydrocarbon gases, displacement of the fluorescent guest was detected, along with fluorescence ‘‘turn-off.” As a later air purge demonstrated, this active F-IDA system was reversible.

Pozo et al. (2018) and Yang et al. (2013) reported an F-IDA for recognizing amantadine (AMA), an acknowledged agent for managing Parkinson’s disease. In the prior report, AMA-induced displacement of a thionine (Th) guest from the cavities of CB7 or CB8 was noted, resulting in a fluorescence attenuation of the CB7 host and a fluorescence intensification of the CB8 host. Yang et al. (2013) adopted a different guest, 1,1′-butane(1,4-diyl)bis(2-aminopyridine)bromide (DPAD), combined with CB7 as the host for detecting AMA by fluorescence quenching. In another study, Liu and co-workers also proposed an F-IDA for recognizing AMA in a highly sensitive and selective way, where CB7 was employed as the macrocyclic host and the fluorescent guest utilized was N-(4-(aminomethyl)benzyl)-1-(anthracen-9-yl)methanamine (ABAM) (Zhu et al., 2018). Powerful binding of ABAM to the CB7 was noted, and ABAM⊂CB7, a fluorescent host–guest mixture, was formed, which sequentially exhibited strong interference resistance and enabled highly selective AMA recognition. The AMA incorporation led to the discharge of ABAM from the CB7 cavity, causing significant fluorescence quenching of the ABAM. It is expected that this system would be profoundly useful for AMA content quantification in innumerable pharmaceuticals and diverse other drug compounds.

In 2015, Li and co-workers reported F-IDAs of CB7 with three fluorophores, palmatine (PAL), berberine (BER), and coptisine (COP), for the detection of both labetalol hydrochloride (LBT) (Li et al., 2015a) and dibucaine (DC) (Li et al., 2015b). Notably, although the labetalol hydrochloride and dibucaine were examined with identical systems, the authors failed to compare the analytes against one another. Later, in 2017, CB7 with the same three fluorophores, PAL, BER, and COP, was also used to detect the anti-cancer drug methotrexate (MTX) through F-IDA (Chang et al., 2017). After the MTX incorporation, linear quenching in the fluorescence intensities was noted for the CB7–PAL, CB7–BER, and CB7–COP. The application of this approach for MTX recognition in plasma samples has been successful, implying its encouraging applicability in diverse practical settings. In 2016, Aryal et al. (2016) reported that certain pharmaceuticals, such as adamantyl-carboxamido-benzenesulfonamide, N-(aminophenyl)-piperidine, and doxorubicin, enabled a naphthalimide (NMI)-derived fluorescent guest from being displaced from the CB7 cavity, and quenching of the fluorescence emission signal was noted.

Lazar et al. (2016) used perylenediimide (PDI-OH), BER, and MB as fluorescent guests for the effective detection of steroidal drugs. Displacement of the fluorescent guest from the cavity of CB8 was caused by a number of steroidal drugs. Recognition of these steroidal drugs at low micromolar levels was possible using these F-IDAs. In 2018, an F-IDA was designed by Wu et al. (2018) for sensing Sunset Yellow (SY) in soft drinks, which exploited the competitive interplays between a CB7 host and signal luteolin/epigallocatechin gallate (EGCG) guests. It was observed that the formation of luteolin⊂CB7 and EGCG⊂CB7 complexes led to the immensely-intensified fluorescence of luteolin and EGCG. The incorporation of SY into either of these two complexes led to extreme fluorescence attenuation. The two above-mentioned fluorescence detection studies are considered to hold broad prospects in food safety and preservation.

In the same year, 2018, a red-NIR F-IDA was reported by Aryal et al. (2018), where a supramolecular CB8–perylene (PMI1) mixture exhibiting an aggregation behavior-induced fluorescence response of “turn-off” in aqueous solutions was utilized. After a stable host–guest mixture was created, the PMI1 was de-aggregated *via* encapsulation to incur a “turn-on” fluorescence response. Due to the addition of strongly affine addictive drugs (as binding guests), PMI1 was displaced from CB8 to again trigger the “turn-off” fluorescence response. With the aid of the PMI1⊂CB8 mixture, successful urinary addictive drug identification was further demonstrated, and the autofluorescence interference with the urine sample was successfully eliminated.

Later, in 2019, Sinn et al. (2019) reported the first CB8-based F-IDA for sensing serum memantine (Mem), an anti-Alzheimer’s drug, utilizing a fluorescent guest methyl pyridine paracyclophane (MPCP) developed for the CB8 host. This allowed serum Mem sensing even within a low range of concentrations, as the host–guest affinities were improved, with *K*_a_ > 10^12^ M^−1^ in water. Later, in 2020, Paudics et al. (2020) reported an F-IDA based on CB7 and 4-(4-(dimethylamino)styryl)-1-phenylpyridiniumiodide (PhSt) for the selective recognition of trimethyl-lysine over other lysine derivatives. Alongside the PhSt binding to CB7, the fluorescence was greatly intensified, suggesting the potential of the CB7–PhSt system as a feasible F-IDA.

Recently, Yan et al. (2022) reported a novel host-guest inclusion complex based on CB8 and PAL, whose AIE behavior in an aqueous solution was outstanding while also demonstrating preferable sensitivity for recognizing ketamine (KET). It was observed that PAL moieties in the CB8 cavity underwent head-to-tail dimeric stacking as a homoternary complex (2:1), which was the origin of the unconventional AIE effects. The incorporation of KET, which exhibited stronger binding to CB8, led to the guest competition-induced PAL displacement from the host cavity and fluorescence regeneration of free PAL.

## Conclusion and outlook

Our current mini-review has attempted to provide a summary of the latest advances and future prospects of F-IDAs that use CB*n* as molecular recognition units. However, CB*n*-based F-IDAs also face some challenges and deficiencies, despite their promising role in molecular sensing. For example, the supramolecular ensembles formed *via* non-covalent interactions during the assays display thermodynamic instability. Due to the variations of equilibrium processes with polarity, viscosity, temperature pH, and concentration, the association constants are greatly impacted. The present comprehensive mini-review, which covers the available literature concerning CB*n*-based F-IDAs, may offer insights and inspirations for developing innovative and improved F-IDAs to remedy extant challenges and deficiencies. Finally, yet importantly, we expect persistent efforts will be devoted to developing more versatile and reliable F-IDAs, so that an ever-broader scope of real-world issues can be addressed, including the detection of the virus responsible for COVID-19 omicron variant.
